# Redox reaction induced Ostwald ripening for size- and shape-focusing of palladium nanocrystals†
†Electronic supplementary information (ESI) available: Experimental procedures, characterization data, and Fig. S1–S5. See DOI: 10.1039/c5sc01787d



**DOI:** 10.1039/c5sc01787d

**Published:** 2015-06-18

**Authors:** Zhaorui Zhang, Zhenni Wang, Shengnan He, Chaoqi Wang, Mingshang Jin, Yadong Yin

**Affiliations:** a Frontier Institute of Science and Technology , State Key Laboratory for Mechanical Behavior of Materials , Xi'an Jiaotong University , Xi'an , Shaanxi 710054 , P. R. China . Email: jinm@mail.xjtu.edu.cn; b Department of Chemistry , University of California , Riverside , California 92521 , USA . Email: yadong.yin@ucr.edu

## Abstract

We report here that size- and shape-focusing can be achieved through the well-known Ostwald ripening process to produce high-quality metal nanocrystals (NCs). Using Pd as an example, we show that the addition of small NCs of appropriate sizes could help in modulating the growth of larger NCs and enable excellent control over both the size and shape uniformity of the products. A detailed mechanistic study showed that the self-focusing of Pd NCs relied on a dissolution and regrowth process induced by redox reaction of HCHO. With the assistance of HCHO, injection of small sacrificial nanocrystals (SNCs), with sizes below a critical value, into larger seeds results in the dissolution of the SNCs and subsequent deposition onto the larger ones, thus allowing the formation of monodisperse Pd NCs. We have identified the critical radius of the SNCs to be ∼5.7 nm for Pd, and verified that SNCs with sizes larger than that could not effectively support the growth of larger seeds. More interestingly, since Ostwald ripening involves matter relocation, this synthetic approach could even break the self-termination growth habits of metal NCs and produce nanocrystals with sizes that are not conveniently accessible by direct growth.

## Abstract 2

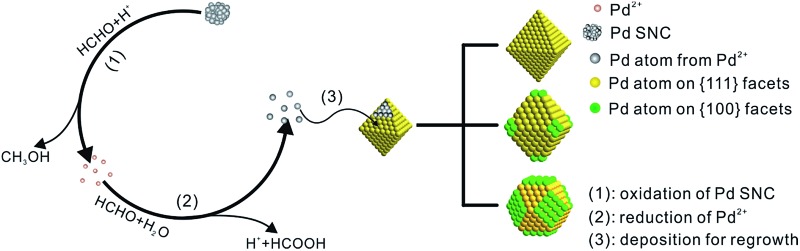
Size- and shape-focusing of palladium nanocrystals have been successfully achieved through the Ostwald ripening process induced by a redox reaction.

## Introduction

Ostwald ripening, a phenomenon where small nanoparticles are dissolved and are re-deposited onto larger particles, has been observed in a number of general nanocrystal (NC) growth systems since first described by Wilhelm Ostwald in 1896.^1^ As a thermodynamically-driven process, Ostwald ripening involves matter relocation and finds applications in many scientific fields, including biology, aerosol science, materials science, surface science, geology, and chemical engineering.^2–6^ Especially in materials science, Ostwald ripening has emerged as an effective synthetic strategy for improving the uniformity of NCs. For example, Johnson *et al.* reported that upconversion NaYF_4_ NCs with a narrow size distribution can be synthesized *via* the epitaxial layer-by-layer ripening-mediated growth method.^7^ Ostwald ripening can also be utilized to achieve inorganic nanomaterials (*e.g.*, TiO_2_, Co_3_O_4_, and Cu_2_O) with hollow interior spaces in solution media.^8–10^ More typically, Ostwald ripening is known as a “size defocusing” process during NC synthesis. One usually needs to precisely control the parameters (such as temperature) to avoid Ostwald ripening so as to obtain NCs with a narrow size distribution. For example, Chen and co-workers have reported an interesting method to prepare monodispersed metal nanocrystals with a clean surface on the substrate through precisely controlling the reaction temperature to avoid ripening.^11,12^ However, Talapin *et al.* recently have shown us a theoretical probable “size focusing” in an ensemble of differently sized NCs unlike the “size defocusing” predicted by the LSW (Lifshitz–Slyozov–Wagner) theory.^13^ In their studies, large particles grow with the consumption of small particles. It produces special-sized (*r*_max_) nanoparticles which have the maximal growth rate. During the Ostwald ripening, small particles dissolve rapidly. At the same time, particles larger than *r*_max_ have growth rates decreasing with *r*, and thus, their size distribution narrows over time, leading to the size-focusing. Later, Peng *et al.* experimentally proved the applicability of the “size-focusing” effect of Ostwald ripening during the growth of MnO NCs.^14^

While it has been more than a decade since these reports, studies on the mechanism and growth kinetics of Ostwald ripening are relatively limited due to the difficulties in separation of the nucleation and growth processes in crystal growth, and the fundamental aspects of such growth, which typically do not follow classical growth mechanisms, have not yet been widely explored. Furthermore, the concept of the critical radius, *r*_b_, has been introduced for a few decades, which separates the smaller sized particles (*r* < *r*_b_), shrinking in size, from the larger particles (*r* > *r*_b_) that became larger with time. When the radius *r* is found to be equal to *r*_b_, the growth is found to be zero.^15,16^ However, the existence of *r*_b_ has not been proved experimentally by far.

In addition, the study of NC growth *via* Ostwald ripening is mostly conducted in the absence of any capping agent, which, when present, inhibits the growth of NCs by effectively passivating specific surfaces. However, the synthesis of almost all NCs is in reality carried out in the presence of a capping agent to stabilize the desired shape for a given application, thereby making the growth process complex. The effect of a capping agent on the modification of the growth kinetics during Ostwald ripening is very specific to the choice of capping agent, therefore, defining the effect of capping agents during the Ostwald ripening process is an important topic, which will subsequently contribute to the rational design and synthesis of NCs.

**Fig. 1:**
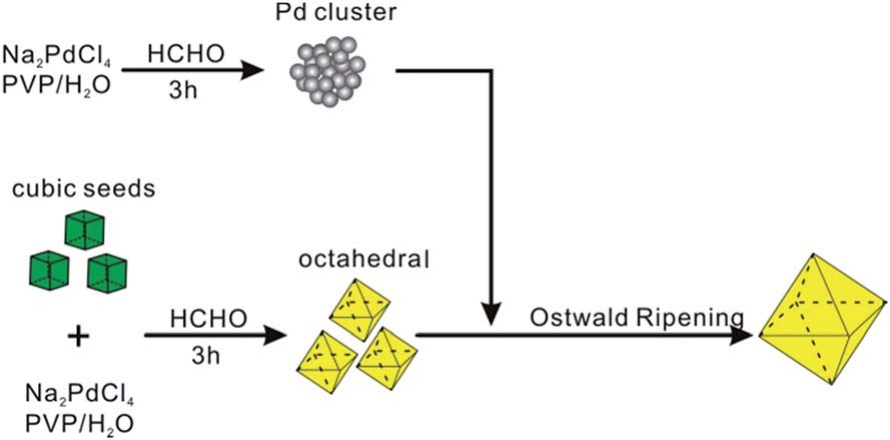
Schematic illustration of Ostwald ripening in a bimodal-sized Pd colloidal system.

Herein, the nucleation and growth processes in crystal growth were separated by employing a bimodal-sized Pd colloidal system in order to investigate the detailed mechanism of the Ostwald ripening process (Fig. 1). As a typical example, formaldehyde (HCHO) was designated to induce a redox reaction, which can act as a carrier for Pd atoms from small sacrificial NCs (SNCs) to larger seeds thus triggering the Ostwald ripening of the Pd NCs. Furthermore, the critical radius of the SNCs, *r*_b_, was able to be validated by experimental methods with the assistance of four types of SNCs with different sizes: 3.2 ± 2.5, 5.0 ± 3.5, 5.7 ± 3.0, and 8.2 ± 2.7 nm. In addition to the study of the ripening process and the critical size, KBr, a specific capping agent for Pd {100} facets, was introduced so as to study the effect of capping agents during Ostwald ripening in this study.

## Results and discussion

Octahedral Pd NCs of 37 nm used in the bimodal-sized Pd colloidal system were synthesized according to our previous work.^17^ Small sacrificial Pd NCs (SNCs) of 5 nm in size were prepared by heating an aqueous solution containing poly(vinyl pyrrolidone) (PVP), HCHO, and sodium tetrachloropalladate (Na_2_PdCl_4_) at 60 °C for 3 h. To ensure that the Pd precursor had been thoroughly reduced to SNCs and that the sequential growth was based upon an internal ripening process, the concentration of [PdCl_4_]^2–^ was characterized by UV-vis spectroscopy after the SNCs formed. As illustrated in Fig. S1,† the absorption peak at 425 nm (ref. 18) that corresponds to [PdCl_4_]^2–^ disappeared after 3 h, indicating the complete reduction of [PdCl_4_]^2–^ by HCHO under the reaction conditions. Then, a 1 mL aqueous solution of Pd octahedra was injected as seeds and allowed to ripen for 3 days before cooling down. The morphological changes at various stages of Ostwald ripening were monitored using transmission electron microscopy (TEM).

**Fig. 2:**
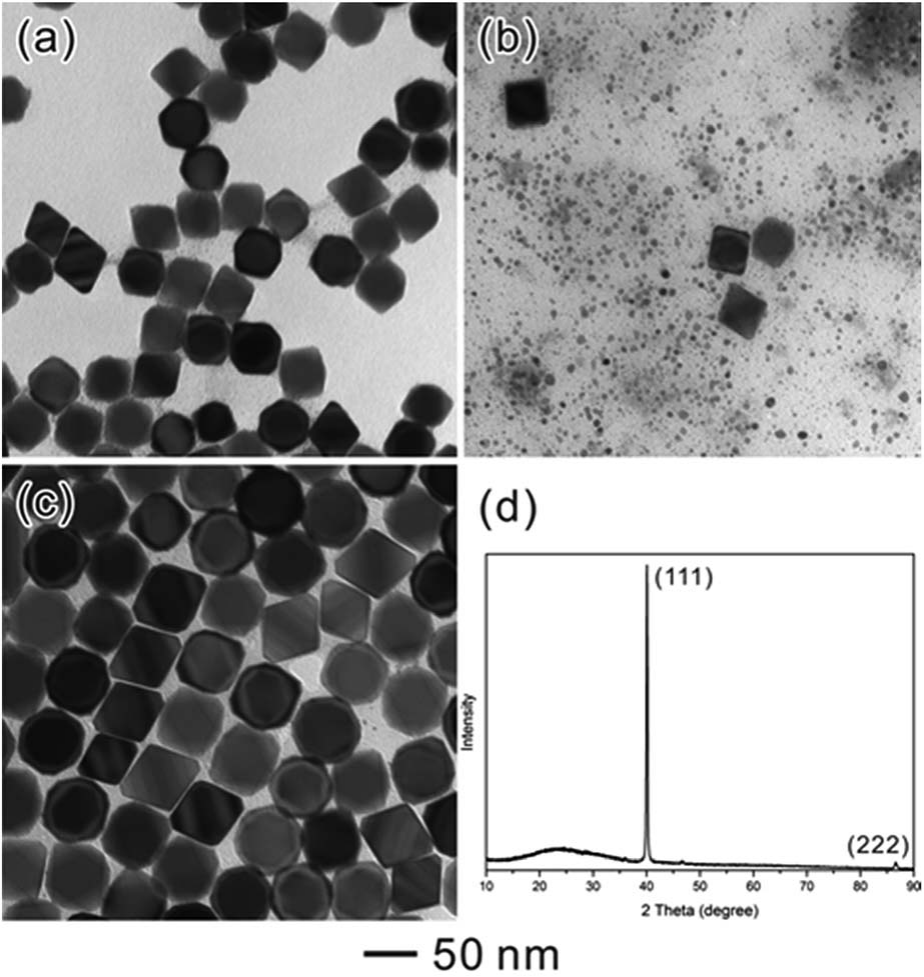
(a–c) TEM images of (a) octahedral seeds, (b) a mixture of seeds with SNCs (*t* = 0 s), and (c) the system after the self-focusing process (*t* = 72 h). (d) XRD pattern of the sample in (c).

Fig. 2a–c show the typical TEM images of three samples at different stages. As can be seen, well-defined octahedral Pd NCs with size around 37 nm have been successfully synthesized and served as larger seeds (Fig. 2a). Fig. 2b shows TEM image of the product after these octahedral Pd seeds were injected into SNCs solution. Clearly, the product exhibits a bimodal size distribution, representing the coexistence of larger Pd octahedral seeds and SNCs at the initial stage (*t* = 0 s). Interestingly, after 3 days' ripening, octahedral Pd seeds of 37 nm were found to grow into monodisperse Pd octahedra with larger size (52 nm). These larger octahedra can easily assemble with their {111} faces lying on the substrate during X-ray diffraction (XRD) analysis. Therefore, the corresponding XRD pattern (Fig. 2d) exhibits a strong (111) peak of Pd, implying a preferential orientation of Pd octahedra, consistent with TEM analyses.

**Fig. 3:**
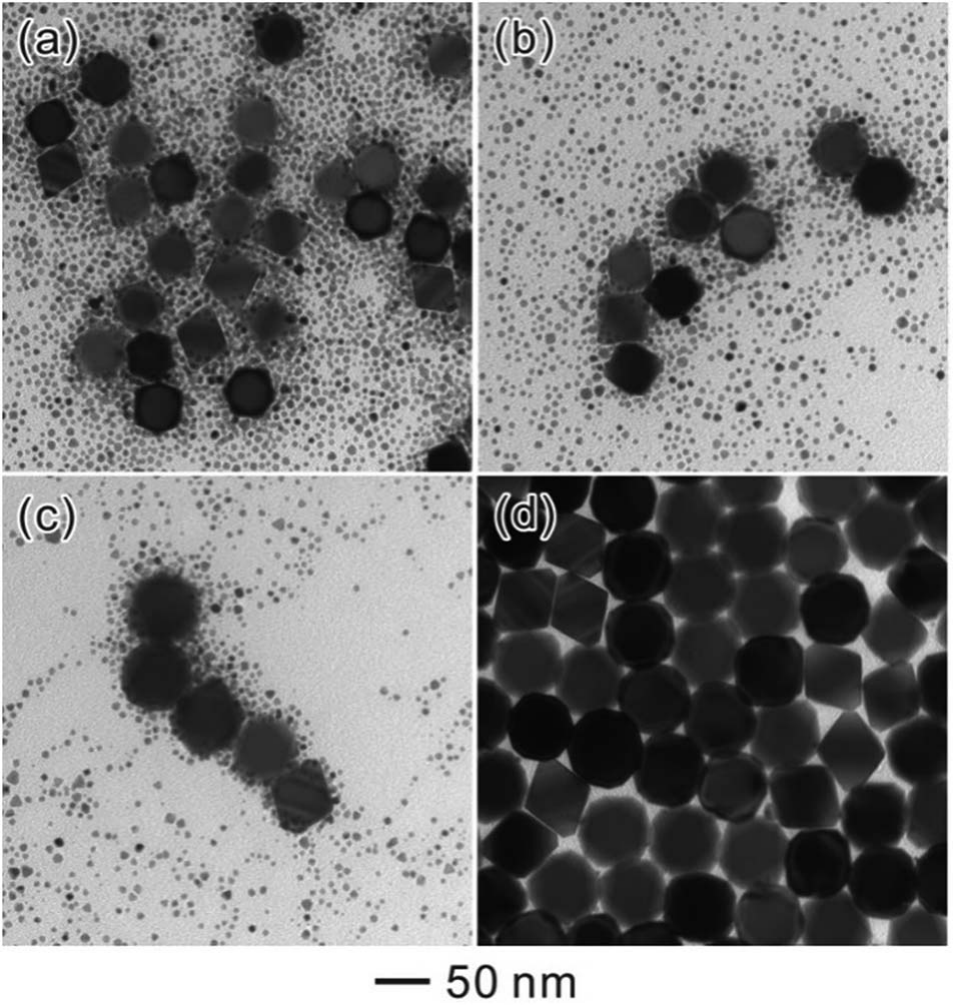
TEM images of Pd octahedra obtained after ripening for different periods of time: (a) 6 h, (b) 24 h, (c) 48 h, and (d) 72 h.

To further understand the growth process of Ostwald ripening, we took TEM images from a set of products sampled from the same reaction solution at different time points (Fig. 3). Obviously, the octahedral seeds grew larger along with the reaction time, while the population of SNCs decreased slowly. These results indicated that Ostwald ripening had taken place, implying the dissolution of SNCs and the growth of octahedral Pd seeds. In order to further identify whether this process was operated by Ostwald ripening or oriented attachment, we substituted the octahedral Pd seeds with quasi-spherical Au NCs (20 nm). As a result, well-defined Au@Pd NCs can be obtained (Fig. S2†) with Au cores located at the center of each NC. This result further proved that Ostwald ripening was the main process because traditional oriented attachment would result in different thicknesses of Pd shells around quasi-spherical Au seeds.

Given that the growth of this bimodal-sized Pd colloidal system was mainly caused by Ostwald ripening, it is fundamentally interesting to elucidate the mechanism: what factor can induce the occurrence of the ripening? As has been widely accepted, the Ostwald ripening process can be separated into two steps, dissolution and re-deposition, both of which may take place simultaneously. This growth is similar to the oxidative etching and regrowth process of metal NCs that was discovered in recent years.^19–23^ As for the oxidative etching process, surface atoms of metal NCs would be oxidized and removed from the NCs by the oxidative etchant, resulting in the formation of metal ions. Almost at the same time, these resultant metal ions would be reduced to atoms and re-deposited onto the added larger seeds, which is known as the regrowth process. It is noteworthy that the oxidizing and reducing agents should be introduced into the reaction system for the oxidative etching and regrowth process. It is likely that the occurrence of Ostwald ripening in our reaction should rely on the presence of oxidizing and reducing agents. However, it is common sense that the reducing and oxidizing agents can't coexist peacefully. Therefore, choosing a special chemical agent that possesses both reducing and oxidizing capabilities is a key factor for Oswald ripening.

As an aldehyde that lack α-hydrogens, HCHO can undergo a disproportionation reaction to give the formation of CH_3_OH and HCOOH under ambient conditions, implying both the oxidizing and reducing abilities of HCHO.^24^ Generally, HCHO has been frequently used to prepare Pd NCs for its reducing capability.^17,25,26^ The reductive reaction is as follows:

1 HCHO + Pd^2+^ + H_2_O ⇋ HCOOH + Pd + 2H^+^

Interestingly, due to the lack of α-hydrogens, HCHO also possesses oxidizing ability, which may induce the oxidative etching process for small Pd nanoparticles under ambient conditions, thus the generation of Pd^2+^ from Pd^0^ (eqn (2)).

2 HCHO + Pd + 2H^+^ ⇋ CH_3_OH + Pd^2+^

**Fig. 4:**
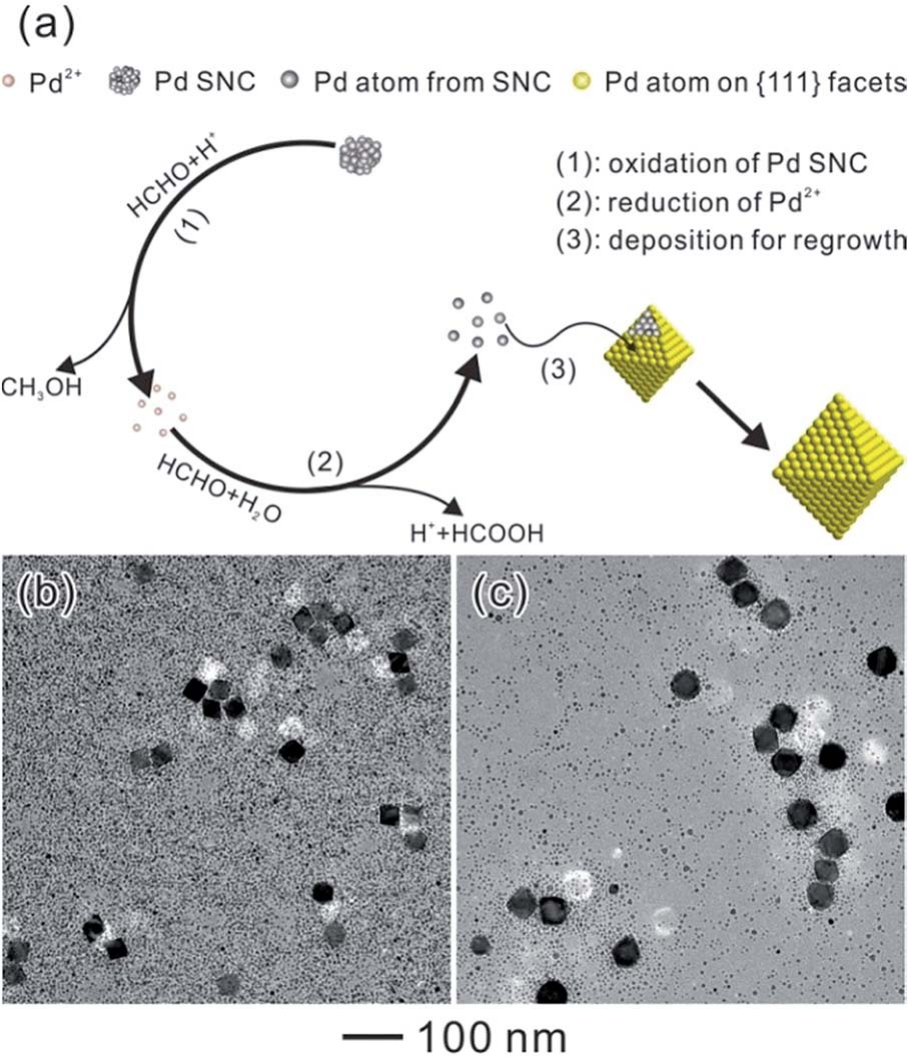
(a) Schematic illustration of the three major steps involved in the oxidative etching and regrowth processes during Ostwald ripening. The redox reactions of HCHO enabled the simultaneous reduction of Pd^2+^ and oxidation of Pd^0^. (b and c) TEM images of the products when the oxidation and reduction process was inhibited by adding extra (b) HCOOH, and (c) CH_3_OH.

As a result, a probable mechanism during the Ostwald ripening of Pd NCs could be illustrated with the key roles played by a specific chemical (HCHO), as shown in Fig. 4a. This ripening process can be divided into three major steps. Because of the oxidizing ability of HCHO and the high energy associated with the SNCs, oxidative etching and the removal of the Pd atoms from the SNCs should start once the octahedral seeds have been injected (step 1). These newly generated Pd^2+^ ions can be reduced back to Pd atoms by HCHO immediately (eqn (1) and step 2), which are then re-deposited onto the octahedral seeds resulting in the formation of larger octahedra (step 3). This process can be proved by detecting the changes in pH and the concentration of CH_3_OH. The initial CH_3_OH concentration is approximately 0 before the addition of Na_2_PdCl_4_, while the pH value is around 3.90. After the addition of Na_2_PdCl_4_, the pH value decreased dramatically from 3.90 to 2.25, implying occurrence of the reduction reaction of Pd^2+^ to Pd^0^ and the formation of the SNCs and HCOOH. The formed SNCs will then be oxidized by HCHO following eqn (2), giving formation of CH_3_OH. Therefore, the concentration of CH_3_OH increased markedly from 0 to 0.657% after the addition of Na_2_PdCl_4_, indicating the existence of an oxidation reaction between the Pd SNCs and HCHO. These newly generated Pd^2+^ ions were then reduced back to atoms *via*eqn (1) and re-deposited onto the larger seeds after the addition of octahedral Pd seeds, accompanied by the further increase in HCOOH concentration and decrease in pH values, as shown in Table 1. A decrease in pH and an increase in the concentration of CH_3_OH can be observed during the reaction, suggesting that HCHO did induce the oxidative etching and regrowth process. In this case, octahedral Pd seeds will grow larger at the expense of the SNCs.

**Table 1:** Relative pH and concentration of CH_3_OH (upper gas) at different ripening times

	*T* ^*a*^ = –3 h	*T* ^*b*^ = 0 h	*T* = 24 h	*T* = 48 h
pH	3.90	2.25	1.83	1.82
CH_3_OH (%)	0	0.657	0.742	0.759

^*a*^Refers to the initial stage before the addition of the Na_2_PdCl_4_ aqueous solution during the synthesis of sacrificial nanocrystals.

^*b*^Refers to the beginning of the ripening-mediated growth.

**Fig. 5:**
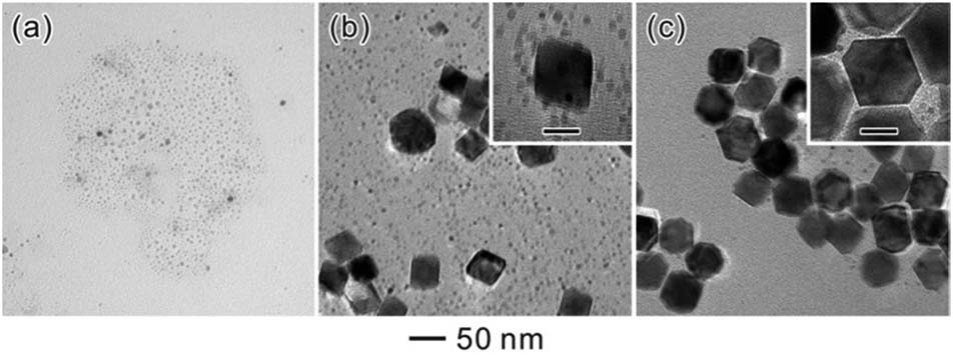
TEM images of Pd (a) SNCs and (b) the final products obtained by using the standard process except for the substitution of HCHO with AA as the reducing agent. The ripening can be recovered by the addition of 100 μL HCHO (c). The insets shows the TEM image of Pd nanocrystals of (b) and (c) at a higher magnification (scale bars: 20 nm).

As shown in eqn (1) and (2), both of the chemical equilibria reflected from these equations can be significantly affected by the addition of HCOOH and CH_3_OH, thus changing the oxidizing and reducing capability of HCHO. Fig. 4b and c show the typical TEM images of products that were prepared under the standard conditions except with the addition of excess HCOOH and CH_3_OH, respectively. The coexistence of residual SNCs is clear and it is observed that the size of the final octahedra significantly decreased, suggesting that the Ostwald ripening has been strongly hindered. Although both of the two inhibitors (HCOOH and CH_3_OH) can hinder the continuity of the ripening-mediated growth of the Pd octahedra, the size in the case of CH_3_OH is slightly larger than in the case of HCOOH. The probable reason is that Pd NCs can also be oxidized by Cl^–^/O_2_, the etchant which has been used to oxidize NCs with twinning or stacking faults,^21^ thus tuning the population of single-crystalline seeds at the initial stage of metal NC synthesis. As the oxidizing ability of Cl^–^/O_2_ is strongly dependent on the environmental oxygen concentrations, we further conducted the Pd bimodal-sized Ostwald ripening reaction in N_2_-purged solutions. Fig. S3† shows the corresponding TEM images. Under N_2_ protection, Ostwald ripening can still take place, and give the formation of Pd octahedra with sizes larger than the original octahedral seeds but smaller than 52 nm. This result indicates that the etching (by Cl^–^/O_2_) can accelerate the Ostwald ripening, but it is not a decisive factor for triggering Ostwald ripening, since Ostwald ripening can still take place under N_2_ protection. In order to further prove the important role of HCHO in inducing the Ostwald ripening of Pd NCs, we employed l-ascorbic acid (AA) as a substituted reducing agent to synthesize SNCs (Fig. 5a). AA has been commonly used as a strong reducing agent for the fast reduction of a Pd precursor,^27^ and it doesn't exhibit any oxidizing ability according to previous reports. Fig. 5b gives the representative TEM images of the product after injecting the octahedral seeds and subsequent 3 days of ripening. Obviously, the product exhibited the same bimodal size distribution as it did at the initial stage. Interestingly, the further addition of HCHO will re-activate the Ostwald ripening, resulting in the ripening-mediated growth of octahedral seeds (Fig. 5c). Therefore, based on the above discussions, a redox reaction induced by HCHO can effectively trigger the Ostwald ripening process, giving the formation of Pd NCs that are highly uniform in size and shape.

Considering the important role of HCHO in triggering the ripening process, it should be expected that the ripening rate can be tuned by changing the concentration of HCHO. In order to elucidate the concentration effect of HCHO, different amounts of HCHO were added (100 μL and 600 μL) to trigger the ripening process. As shown in Fig. S5,† it is clear that the growth of the octahedra is significantly accelerated, implying a higher ripening rate at a higher concentration of HCHO. In addition to the concentration of HCHO, the reaction temperature is of great importance in tuning the rate of the ripening process, too. As reflected by the growth rate of the octahedra (Fig. S6†), obviously, the rate of the ripening process increases along with increasing temperatures. Both of these results indicate that the ripening rate can be easily controlled by varying the reaction conditions, including the concentration of HCHO, and the reaction temperature.

The Ostwald ripening process usually possesses the feature where larger particles grow at the expense of smaller ones due to the Kelvin effect.^28^ According to the Kelvin effect small nanoparticles have a higher solubility, and the corresponding equation of the critical radius is as follows:^29^

**Fig. 6:**
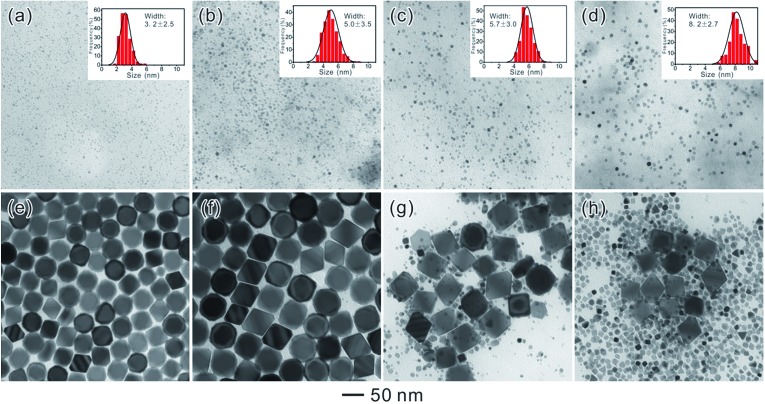
(a–d) TEM images of four SNC samples with different particle sizes obtained by controlling the amount of Na_2_PdCl_4_ in the reaction: (a) 14.5 mg, (b) 29 mg, (c) 57 mg, and (d) 114 mg. The inset in each panel shows the size distribution of the SNCs. (e–f) Corresponding products after 3 days of ripening.

3 *C*(*r*) = *C*(∞)exp(*α*/*r*)

where *C*(*r*) is the solubility of a dispersed phase particle with radius *r*. The bulk solubility *C*(∞) corresponds to the solubility of a particle with an infinite radius, that is, to the solubility of the dispersed phase when it has a flat surface or the bulk solubility; *α* is called the capillary length. It can be seen that small nanoparticles are more soluble than large ones. Thus the larger nanoparticles can capture the monomers released from the smaller ones resulting in an increase in their size. It should have a critical radius to reach equilibrium. Hence, validating this assumption by experiments would be highly desired. On the basis of four types of SNCs with different sizes, we designed a set of experiments to demonstrate the accurate value of the “critical radius”. Four types of Pd SNCs with different sizes (3.2 ± 2.5, 5.0 ± 3.5, 5.7 ± 3.0, and 8.2 ± 2.7 nm) were synthesized by controlling the amount of Na_2_PdCl_4_ added to the reaction solution, and used as SNCs for the further Ostwald ripening reaction (Fig. 6a–d). Fig. 6e–h show the final products after Ostwald ripening. For SNCs with average sizes of less than 5.7 nm, the octahedral seeds grew into monodisperse larger ones, indicating that Ostwald ripening (Fig. 6e and f) has taken place and that the SNCs dissolved and re-deposited onto larger seeds. When the average size of the SNCs introduced into the synthesis was larger than 5.7 nm, re-deposition will occur on both of the SNCs (larger than 5.7 nm) and octahedral seeds.

Therefore, 5.7 nm should be a critical size for the Ostwald ripening of Pd NCs in our reaction. During the Ostwald ripening of Pd NCs, SNCs with a size less than 5.7 nm will be oxidized, dissolved, and re-deposited onto larger seeds. Accordingly, another decisive factor for inducing Ostwald ripening to achieve monodisperse Pd NCs with well-defined shapes and sizes is to use SNCs with sizes smaller than 5.7 nm.

**Fig. 7:**
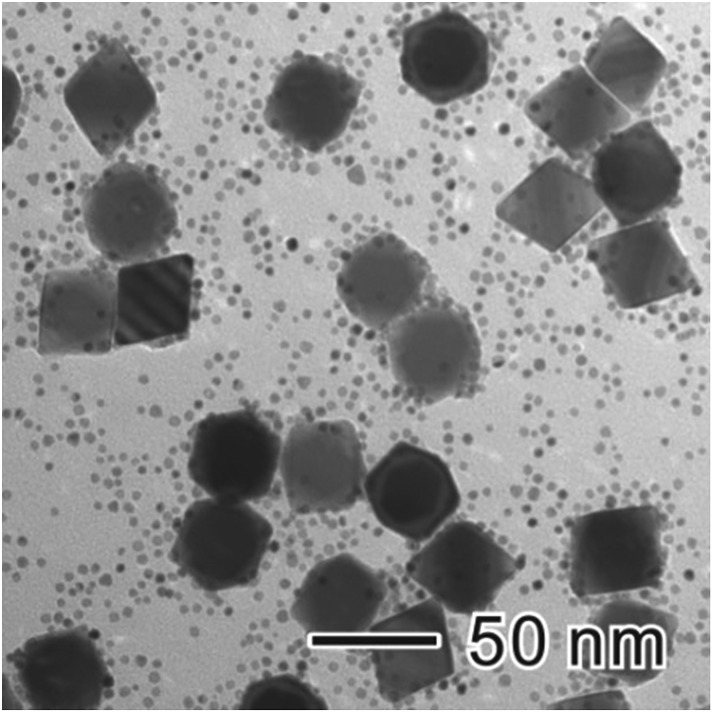
TEM image of Pd nanocrystals prepared with 57.0 mg of Na_2_PdCl_4_. In addition to Pd octahedra of 37 nm in edge length, small Pd nanoparticles of 3 nm in size also formed. In this case, simply increasing the amount of Pd precursor did not increase the size of the large Pd octahedra.

It is well-established that the strong and preferential adsorption of capping agents toward specific facets has enabled us to tailor the surface structure of metal NCs. However, this strong adsorption, on the other hand, would tend to hinder the further growth of metal NCs. Especially for those metal NCs with octahedral shapes (exposed with the most stable {111} facets), a large energy barrier is required to be overcome for newly formed atoms to deposit onto their surfaces. Therefore, it is difficult to prepare octahedral NCs with different sizes *via* seeded growth. Such as in the growth of Pd cubic seeds, one can only obtain octahedra of a single size, which was roughly 2.1 times of the edge length of the cubic seed (Fig. S4†). After reaching this point, the growth was automatically terminated, and further increasing of the amount of Pd precursor did not favour a size increasing (Fig. 7). Instead, the final product became a mixture of Pd octahedra and small Pd nanoparticles.

This phenomenon is named self-termination growth, and has also been observed in the seeded growth of many other metal NCs such as Ag, in which it strongly limited their size-tunability.^30,31^ Thus it is necessary to select an appropriate method to overcome this self-termination. As Ostwald ripening involves matter relocation, it may provide the possibility of breaking the “self-termination” growth habit of metal NCs. Based on a clear understanding of the mechanism of Ostwald ripening, this versatile ripening-mediated growth could be extended to obtain metal NCs with controlled sizes and shapes.

**Fig. 8:**
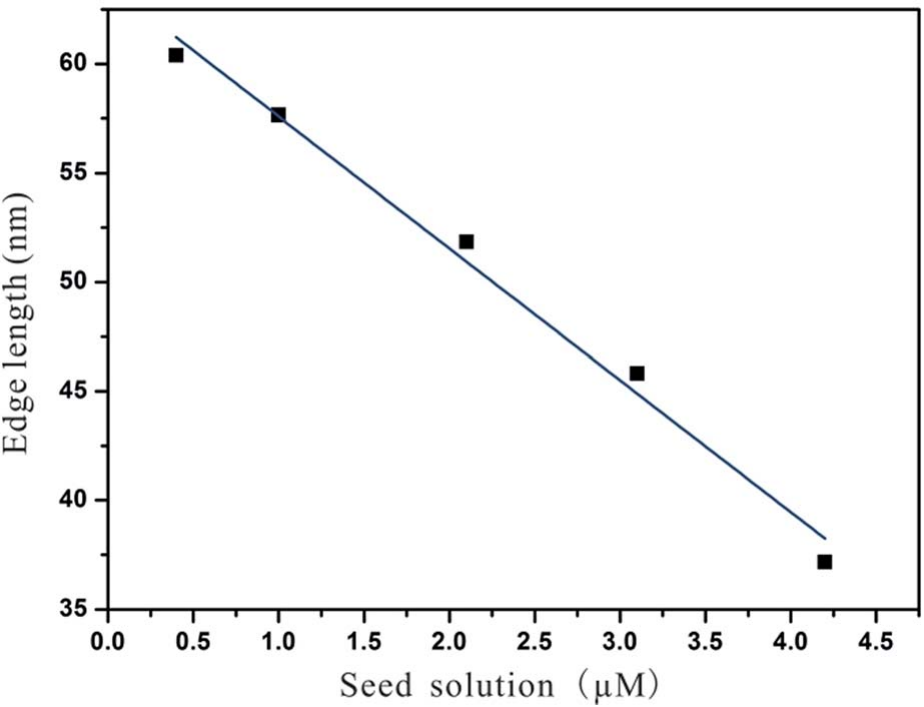
Decrements in edge length with the calculated seed concentration of 0.4, 1.0, 2.1, 3.1, and 4.2 μM.

**Fig. 9:**
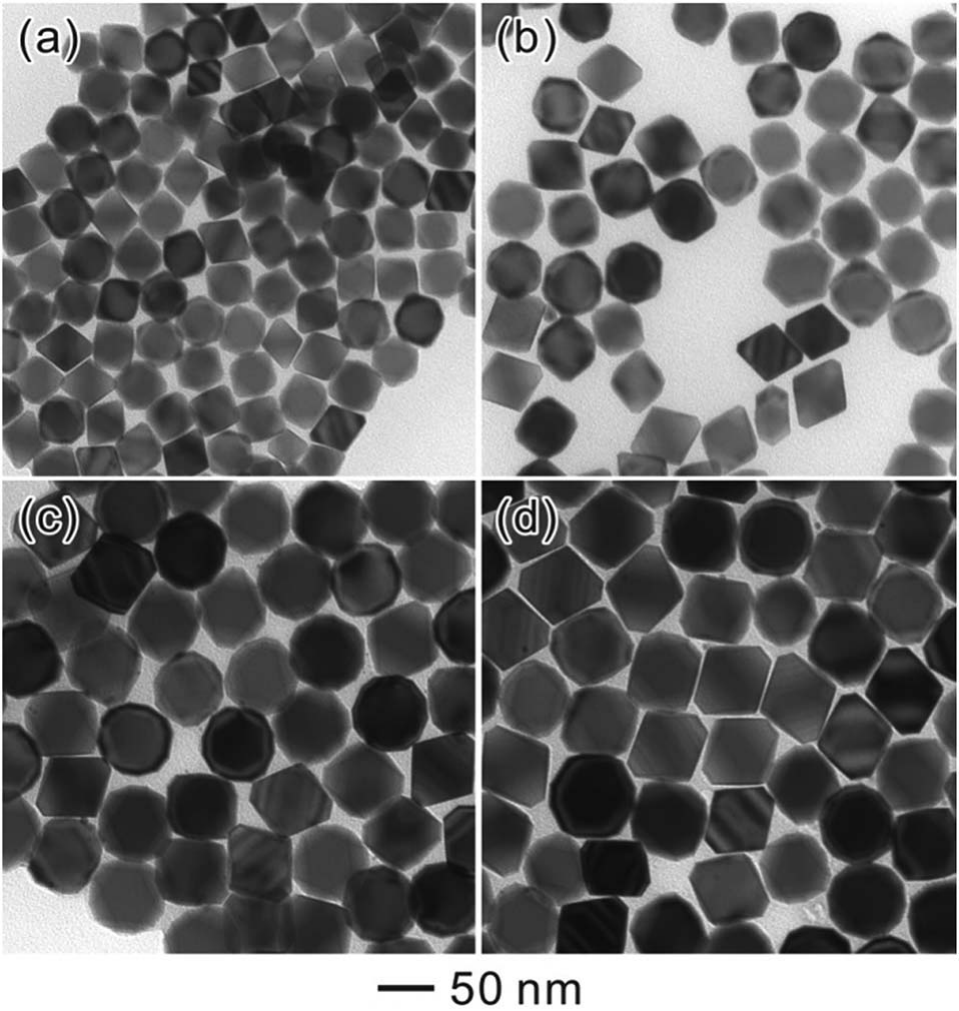
TEM images of Pd octahedra with different edge lengths that were prepared using the standard procedure except with different concentrations of Pd seeds: (a) 4.2 μM, (b) 3.1 μM, (c) 1.0 μM, and (d) 0.4 μM respectively.

By simply varying the concentration of seeds, self-termination growth of Pd can be easily broken and the size of the final products could be readily controlled from approximately 37 nm to 60 nm in edge length. As shown in Fig. 8 and 9, it can be seen that the edge lengths of the resultant Pd octahedra increased from 37 to 46, 52, 58 and 60 nm linearly when the concentration of Pd seeds was reduced from 4.2 to 3.1, 2.1, 1.0, and 0.4 μM. Thus we can conclude that Ostwald ripening could break the self-termination growth habit of Pd NCs discovered recently and prepare Pd octahedra with sizes ranging from 37 to 60 nm. It should be pointed out that the octahedra shown in Fig. 9 exhibited a truncation at the corners due to the selective adsorption of PVP onto the Pd {100} facets when the size of the Pd NCs is larger than 50 nm.^32^ This selective capping will reduce the growth rate along 100 and eventually lead to the formation of Pd octahedra with a slight truncation at the corners.

**Fig. 10:**
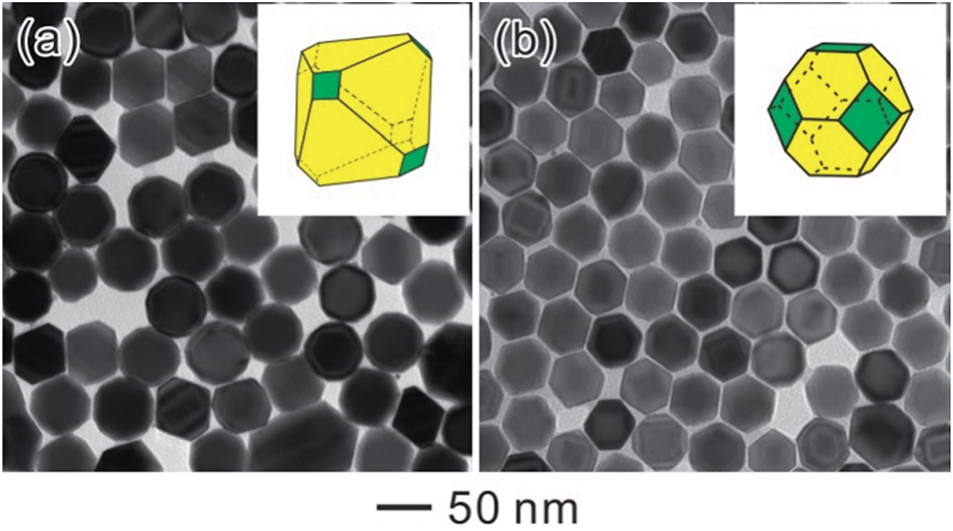
TEM images of Pd NCs obtained by the ripening-mediated growth in the presence of different amounts of KBr: (a) 300 mg, (b) 400 mg.

Accordingly, Ostwald ripening can also be combined with the capping effect for the shape-controlled synthesis of Pd NCs. Fig. 10 shows typical TEM images of the resultant Pd NCs evolved from the octahedral seeds by introducing KBr as a capping agent. Specifically, truncated octahedra were obtained when KBr was used at an amount of 300 mg (Fig. 10a). Further increasing the amount of KBr to 400 mg led to the formation of cuboctahedra (Fig. 10b). As we noted earlier, Br^–^ ions could selectively adsorb onto the Pd {100} facets to increase the energy barrier for further deposition and reduce the growth rate along the 100 direction.^27^ However, Br^–^ ions could also coordinate with the Pd^2+^ ions to form a more stable ionic complex, PdBr_4_^2–^, to decelerate the reduction rate since it is more difficult to reduce PdBr_4_^2–^ into Pd atoms relative to Pd^2+^.^33,34^ When the concentration of Br^–^ ions in the growth solution was low, only a small portion of the Pd^2+^ ions, generated in step 1 (Fig. 4a), could be converted into the PdBr_4_^2–^ complex. The reaction rate of step 2 (Fig. 4a) remained fast. However, when the concentration of Br^–^ ions exceeded a critical point, the energy barrier for further deposition of Pd atoms onto the octahedral seeds would be higher than that for self-nucleation. In this case, irregular-shaped Pd nanoparticles and decahedra can be obtained (Fig. S7†).

## Conclusions

In summary, we demonstrated that the Ostwald ripening process triggered by a redox reaction could be used to modulate the growth of Pd NCs and improve the control over the uniformity of their size and shape. A critical size was identified, ∼5.7 nm for Pd, above which substantial ripening-mediated growth would not occur readily. Capping ligands were also found be able to contribute to the shape evolution during the ripening-mediated growth. In addition to enhancing particle uniformity, the versatile ripening-mediated growth strategy also has the advantage of breaking the self-termination growth habits of Pd NCs and produce Pd NCs with sizes not easily available by conventional direct synthesis. The results reported here suggest that Ostwald ripening may serve as an alternative but still general approach for effectively controlling the growth behavior of various nanocrystals.
