# Supplementary material for: Redox reaction induced Ostwald ripening for size- and shape-focusing of palladium nanocrystals
Source: Chem Sci. 2015 Jun 18;6(9):5197–203. doi: 10.1039/c5sc01787d (PMC5669216; doi:10.1039/c5sc01787d)
Supplement: Supplementary file 1 [file SC-006-C5SC01787D-s001.pdf]

Electronic Supplementary Material (ESI) for Chemical Science.
This journal is © The Royal Society of Chemistry 2015

Supporting Information

Redox Reaction Induced Ostwald Ripening for Size- and Shape-Focusing of Palladium Nanocrystals

Zhaorui Zhang,[a] Zhenni Wang,[a] Shengnan He,[a] Chaoqi Wang,[a]

Mingshang Jin,[*a] and Yadong Yin,[*b]

[a]Center for Materials Chemistry, Frontier Institute of Science and Technology, Xi'an Jiaotong University, Xi'an, Shaanxi, 710054, P. R. China

[b]Department of Chemistry, University of California, Riverside, California 92521, USA.

MATERIALS AND METHODS

Materials.

L-ascorbic acid (AA, > 99%), poly(vinyl pyrrolidone) (PVP, Mw = 55000, 10000), sodium tetrachloropalladate(II) (Na_2PdCl_4, 98%), potassium iodide (KI, > 99%), Gold (III) chloride ($HAuCl_4$, > 99%), formaldehyde solution (HCHO, 37%), formic acid (HCOOH, 98%), methanol (CH_3OH, >99.9%), and potassium bromide (KBr, > 99%) were all obtained from Sigma–Aldrich and used as received. Another formaldehyde solution (HCHO, 10.0 mg/mL, aqueous solution without any stabilizer) was purchased from Aladdin. Deionized water with a resistivity of 18.2 MΩ·cm was used for the preparation of all aqueous solutions.

Syntheses of sacrificial NCs with different sizes.

In a standard synthesis, an aqueous solution (7 mL) that contained 105 mg PVP (Mw = 55000) and 100 μL HCHO was heated at 60 ºC in a 20 mL vial under magnetic stir-ring. After 5 min, 3.0 mL of an aqueous solution containing Na_2PdCl_4 at various amount (14.5, 29, 57, and 114 mg, respectively) was rapidly injected into the vial using a pipette. The reaction lasted for 3 h, and the resultant solution was stored and used as a stock solution for the further growth. To investigate the influence of HCHO on the ripening-mediated epitaxial growth, 100 μL HCHO was replaced by 20 mg AA while the total volume of the reaction mixture and concentration of the precursor were remained the same.

Synthesis of 37-nm Pd octahedral seeds.

The 37-nm Pd octahedral seeds were synthesized based on a previously reported procedure by our group.[1] In a typical synthesis, 3.0 mL of an aqueous solution containing Na_2PdCl_4 (29 mg) was introduced into 8 mL of an aqueous solution containing 105 mg PVP (Mw = 55000), 100 μL HCHO, and 0.3 mL of an aqueous suspension (1.8 mg/mL in concentration) of Pd cubic seeds[2] 18 nm in edge length, which had been heated at 60 ºC for 5 min under magnetic stirring in a capped vial. The reaction was allowed to proceed at 60 ºC for 3 h, and the resultant Pd seeds were collected and washed by centrifugation. Finally, the seeds were redispersed in deionized water (1 mL) for storage and further use.

Synthesis of quasi-spherical Au seeds.

A seeded-growth route was chosen for large-scale synthesis of Au nanoparticles of a predetermined size.[3] Typically, a growth solution was prepared by incorporating 12 mL of PVP (Mw = 10 000), 6 mL of AA (0.1 M), 4.5 mL of KI (0.2 M), and 1.8 mL of $HAuCl_4$ (0.254 M) in 60 mL of H_2O. 16 mL of a seed solution (3.5 nm Au nanoparticles), which was prepared according to a reported literature was quickly injected into this growth solution.[4] After 10 mins' reaction, the quasi-spherical Au nanoparticles formed and were collected and washed by centrifugation, and then redispersed in 120 mL of H_2O as a stock solution.

Ripening-mediated epitaxial growth.

The synthesis of the SNCs was done as described above, and after 3 h' reaction at 60 ˚C, 1 mL of 37-nm Pd octahedral seeds was injected into the reaction mixture (t = 0) and allowed to ripen for 3 days (t = 72 h). After that, the product was collected by centrifugation, and then washed with water twice. The product was finally re-dispersed in ethanol for further characterization. To verify the concept of the "redox induced dissolution and regrowth" process, 100 μL HCOOH or CH_3OH was added at t = 0 followed by the injection of the Pd octahedral seeds. To obtain Pd NCs with

different sizes, the amount of Pd seeds was varied. To obtain Pd NCs with different shapes, different amount of KBr was added at t = 0 followed by the injection of the Pd octahedral seeds.

Synthesis of Au@Pd NCs.

The synthesis was carried out exactly as outlined for Pd NCs mentioned above, except that the 1 mL of 37-nm Pd octahedral seeds was replaced by 1 mL quasi-spherical Au seeds.

Methanol detection.

To eliminate the influence of methanol in the HCHO (Sigma–Aldrich), the reaction was carried out under the same condition as the standard except that HCHO (Sigma–Aldrich) was replaced by HCHO (Aladdin). Then the gases above this solution were taken at different times for GC-MS measurements.

Characterizations.

Transmission electron microscopy (TEM) analysis was performed with a Hitachi HT-7700 electron microscope equipped with a tungsten filament, operating at 100 kV. UV−vis extinction spectra were measured on an Ocean Optics HR2000+ES UV−vis−NIR spectrophotometer with a DH-2000-Ballight source. GC-MS analysis was performed on an Agilent 7890A GC system equipped with an Agilent 5975C mass-selective detector and an HP-5 MS capillary column. Powder XRD patterns were recorded using a diffractometer (X-ray Diffractometer SmartLab(3), Rigaku) operated at 3 kW.

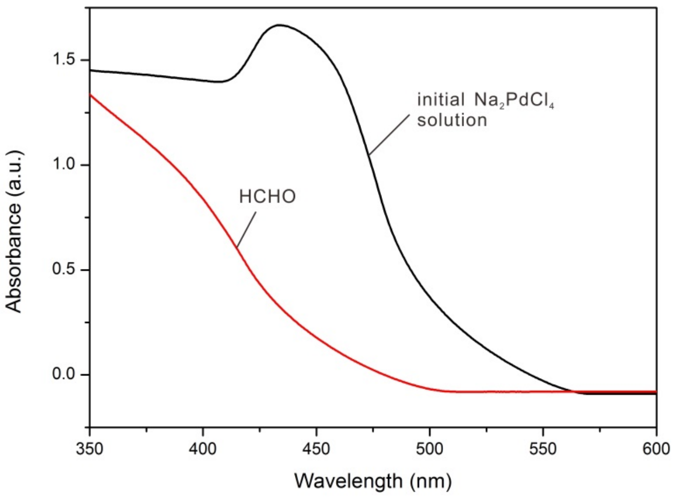

Figure S1. UV-vis spectra from tests on 8.7 mM Na_2PdCl_4 solutions containing 100 µl HCHO as a reducing agent after heating for 3 h at 60 °C; This condition corresponds to that for the syntheses of Pd SNCs. The absorption peak at 425 nm is directly proportional to the concentration of the $[PdCl_4]^{2-}$ species.

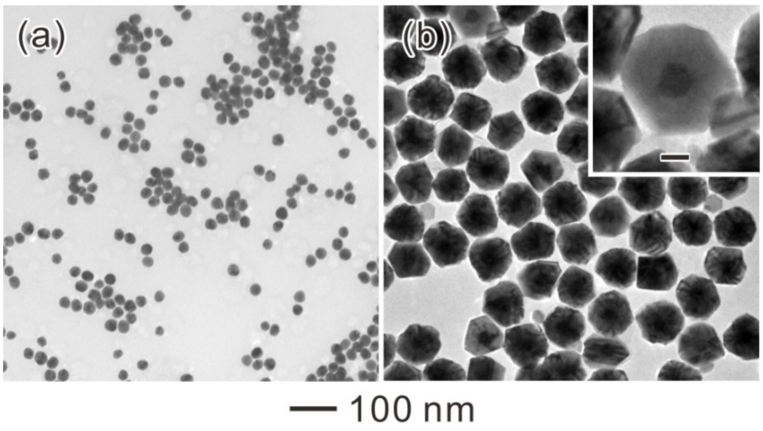

Figure S2. TEM images of (a) spherical Au seeds, and (b) corresponding ripening products. The inset in (b) shows the TEM image of Au@Pd nanocrystals at a higher magnification (scale bars: 20 nm).

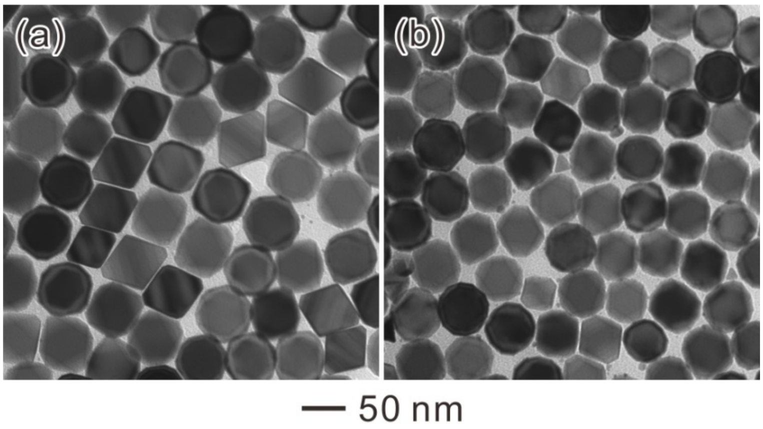

Figure S3. TEM images of the final products when the ripening was conducted in: (a) air, and (b) N_2 gas.

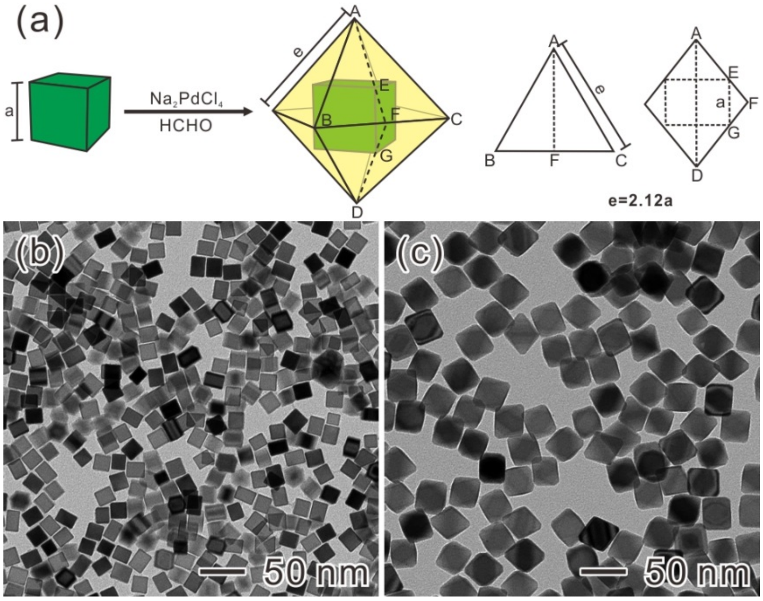

Figure S4. (a) Schematic illustrating the seed-mediated growth of Pd octahedra from single-crystal cubic seeds of Pd. The size "*e*" of the resultant Pd octahedra is determined by the size "*a*" of the cubic seed. TEM images of Pd cubes (b) 18 nm in edge length and (c) the corresponding octahedrons grown from this cubic seeds.

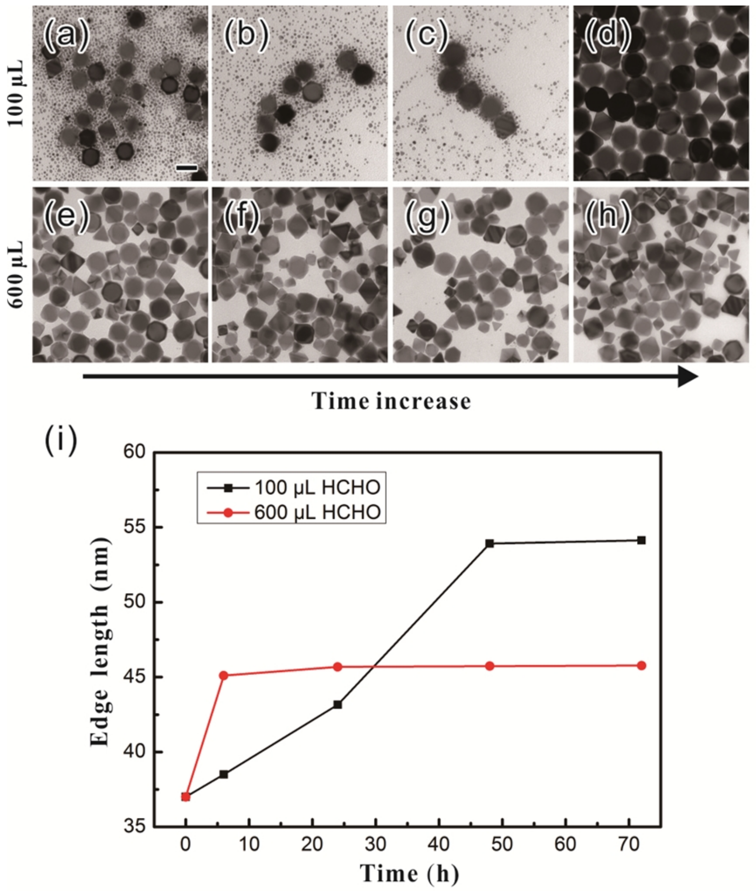

Figure S5. TEM images of Pd octahedra obtained after ripening for different periods of time at typical condition (a-d), and with additional HCHO (e-h). (I) Changes of final size as a function of the ripening time. (scale bars: 50 nm)

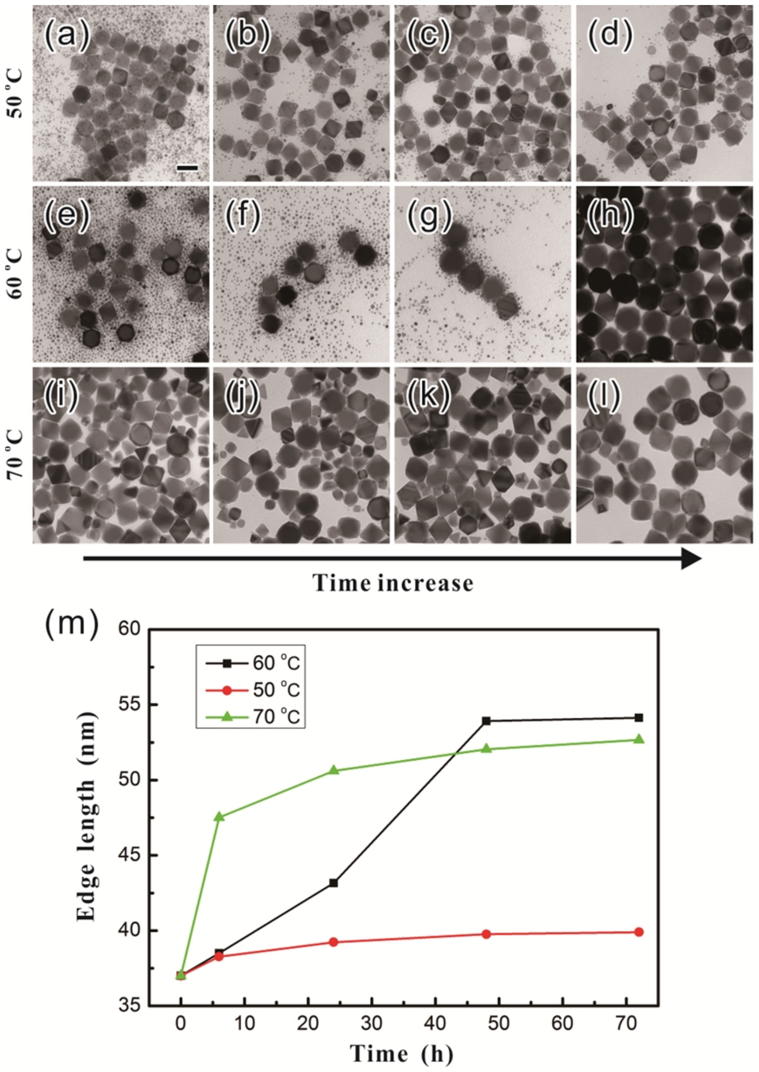

Figure S6. TEM images of Pd octahedra obtained after ripening for different periods of time at 50°C (a-d), 60°C (e-h) and 70°C (i-l). (m) Changes of final size as a function of the ripening time. (scale bars: 50 nm)

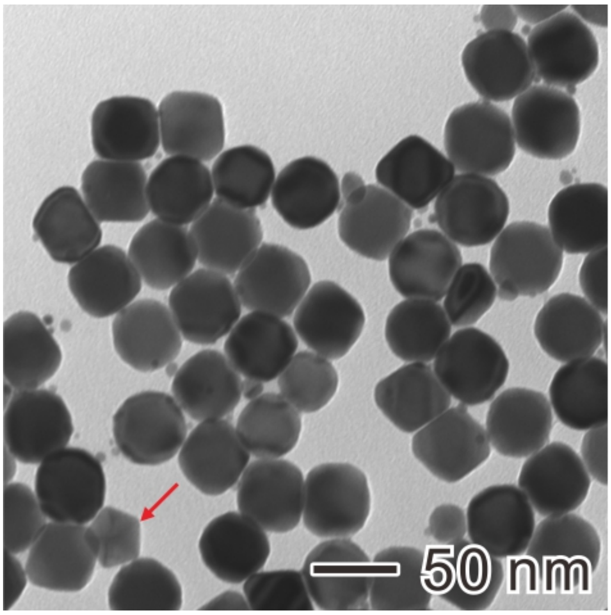

Figure S7. TEM image of Pd nanocrystals obtained by the ripening-mediated growth in the presence of 600 mg KBr. The red arrows indicate the decahedra generated during the ripening.

References

1 M. Jin, H. Zhang, Z. Xie, and Y. Xia, *Energy Environ. Sci.* **2012**, *5*, 6352.
2 M. Jin, H. Liu, H. Zhang, Z. Xie, J. Liu, and Y. Xia, *Nano Res*. **2011**, *4*, 83.
3 C. Gao, J. Vuong, Q. Zhang, Y. Liu, and Y. Yin, *Nanoscale* **2012**, *4*, 2875.
4 N. R. Jana, L. Gearheart, and C. J. Murphy, *Langmuir* **2001**, *17*, 6782.
